# Hydrophobic Substituents of the Phenylmethylsulfamide Moiety Can Be Used for the Development of New Selective Carbonic Anhydrase Inhibitors

**DOI:** 10.1155/2014/523210

**Published:** 2014-09-02

**Authors:** Giuseppina De Simone, Ginta Pizika, Simona Maria Monti, Anna Di Fiore, Jekaterina Ivanova, Igor Vozny, Peteris Trapencieris, Raivis Zalubovskis, Claudiu T. Supuran, Vincenzo Alterio

**Affiliations:** ^1^Istituto di Biostrutture e Bioimmagini, CNR, Via Mezzocannone 16, 80134 Naples, Italy; ^2^Latvian Institute of Organic Synthesis, Aizkraukles 21, Riga LV-1006, Latvia; ^3^Laboratorio di Chimica Bioinorganica, Università degli Studi di Firenze, Room 188, Via della Lastruccia 3, 50019 Sesto Fiorentino, Florence, Italy; ^4^Dipartimento di Scienze Farmaceutiche, Università degli Studi di Firenze, Polo Scientifico, Via Ugo Schiff 6, 50019 Sesto Fiorentino, Florence, Italy

## Abstract

A new series of compounds containing a sulfamide moiety as zinc-binding group (ZBG) has been synthesized and tested for determining inhibitory properties against four human carbonic anhydrase (hCA) isoforms, namely, CAs I, II, IX, and XII. The X-ray structure of the cytosolic dominant isoform hCA II in complex with the best inhibitor of the series has also been determined providing further insights into sulfamide binding mechanism and confirming that such zinc-binding group, if opportunely derivatized, can be usefully exploited for obtaining new potent and selective CAIs. The analysis of the structure also suggests that for drug design purposes the but-2-yn-1-yloxy moiety tail emerges as a very interesting substituent of the phenylmethylsulfamide moiety due to its capability to establish strong van der Waals interactions with a hydrophobic cleft on the hCA II surface, delimited by residues Phe131, Val135, Pro202, and Leu204. Indeed, the complementarity of this tail with the cleft suggests that different substituents could be used to discriminate between isoforms having clefts with different sizes.

## 1. Introduction

Carbonic anhydrases (CAs, EC 4.2.1.1) are ubiquitous metalloenzymes found in prokaryotes and eukaryotes, which catalyze the reversible hydration of carbon dioxide to bicarbonate ion and proton (CO_2_ + H_2_O⇆HCO_3_^−^ + H^+^) [1, 2]. In humans 15 different isoforms have been identified so far, among which 12 are catalytically active (CAs I-IV, VA-VB, VI-VII, IX, and XII-XIV), whereas the remaining three (CAs VIII, X, and XI), named as CA-related proteins (CARPs), are devoid of any catalytic activity [2]. All the catalytically active isoforms contain in their active site a zinc ion tetrahedrally coordinated by three conserved histidine residues and a water molecule/hydroxide ion [1, 2].

**Figure 1:**
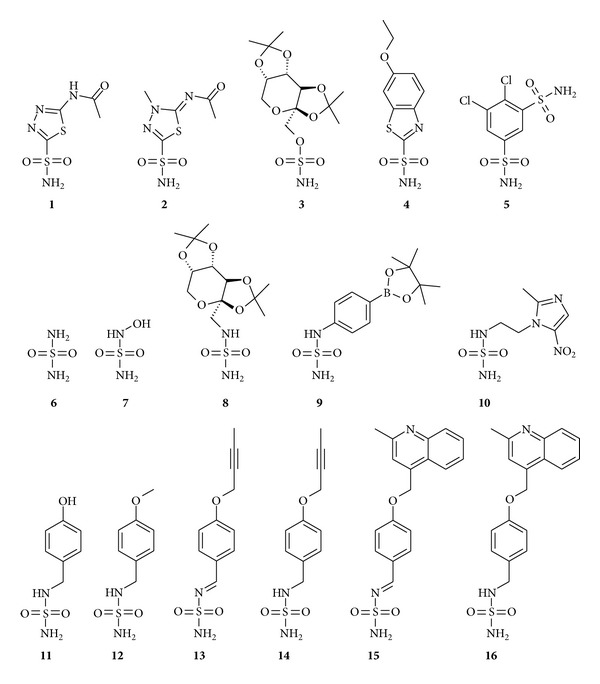
Chemical formulas of inhibitors** 1**–**16**.

Over the past few years, the discovery of the involvement of several CA isoforms in human diseases has greatly increased the attention on these enzymes in regard to their consideration as interesting targets for drug design [3]. Indeed, a wealth of derivatives, mainly containing a primary sulfonamide (RSO_2_NH_2_) [1, 2, 4–6] and its bioisosteres, such as the sulfamate (ROSO_2_NH_2_) [1, 7, 8] and sulfamide (RNHSO_2_NH_2_) [1, 2, 9–18] as zinc anchoring groups, have been investigated as CA inhibitors (CAIs) with some of them (principally sulfonamides and sulfamates) being explored for the treatment of a variety of disorders such as glaucoma [19–22], acid-base disequilibria [23], epilepsy [24, 25] neuromuscular diseases [26], edema [27], and obesity [28, 29] and for the management of hypoxic tumors [30]. Acetazolamide (AAZ)** 1** [31], methazolamide (MZA)** 2** [31], topiramate (TPM)** 3** [32], ethoxzolamide (EZA)** 4** [33], and dichlorphenamide (DCP)** 5** [31] represent some examples of such pharmacologically relevant CAIs (Figure 1). However, it is important to highlight that none of the currently clinically used CAIs shows selectivity for a specific isozyme [1].

The knowledge of the inhibition profile of CAIs against all human isoforms and of their detailed binding to the enzyme (which can be obtained from crystallographic data) can allow for a better understanding of their mechanism of action and can provide an efficient molecular basis for the rational drug design of isozyme-selective compounds [1, 34]. In the last decade a huge number of X-ray structural studies of CA adducts principally with sulfonamides and sulfamates have been reported. On the contrary, sulfamide-containing derivatives have been only poorly investigated as CAIs, because they were initially supposed not to be particularly suitable for obtaining potent CA inhibitors, exhibiting just a moderate-to-weak inhibition potency [35, 36]. However, many recent studies, predominantly by Supuran's group, have supported the idea that sulfamide derivatives can be considered interesting candidates for obtaining CAIs, showing such several compounds with relatively high CA affinity [12–15]. At present, only 5 sulfamide-containing derivatives have been characterized by means of X-ray crystallography for their interaction with CAs: the simple sulfamide** 6** [9, 16], the N-hydroxy-sulfamide** 7** [10, 18], the sulfamide derivative of the antiepileptic drug topiramate** 8** [11–15, 17], the boron containing derivative** 9** [37], and the nitroimidazole-sulfamide** 10** [38] (Figure 1). Thus we decided to investigate in more detail this class of inhibitors by means of kinetic and crystallographic studies. In particular, in this paper we describe the synthesis and the inhibition analysis of a series of new sulfamides (compounds** 11**–**16**) with CA isoforms I, II, IX, and XII. Furthermore, to better understand at structural level the molecular features determining the inhibition profiles of such compounds, we also report the high-resolution crystallographic structure of the cytosolic dominant isoform hCA II in complex with the highest affinity inhibitor (compound** 14**) in the newly synthesized series.

## 2. Results and Discussion

### 2.1. Chemistry

**Scheme 1:**
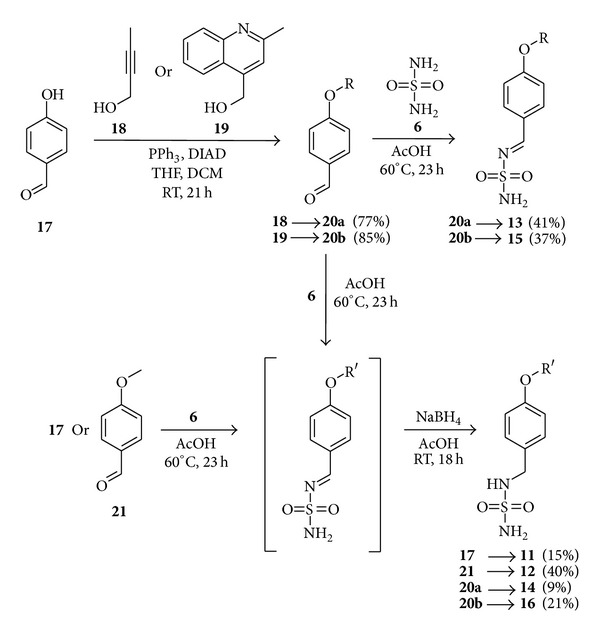
Synthesis of compounds** 11**–**16**.

Synthesis of aza-benzylidene derivatives of sulfamide, like compound** 13**, from aryl aldehydes and sulfamide, is reported in the patent literature [39]. Our first efforts to reproduce a published procedure where ethanol was used as a solvent resulted in formation of trace amounts of desired product. After screening of several solvents we found that the use of glacial acetic acid gave reproducible results. With the improved procedure, where equimolar amounts of aryl aldehydes (compounds** 20a-b**) and sulfamide (compound** 6**) were used, monosubstituted aza-benzylidene derivatives** 13** and** 15** were isolated in acceptable yields (Scheme 1). Substituted aryl aldehydes** 20a-b** were prepared from 4-hydroxybenzaldehyde** 17** and corresponding alcohols** 18** or** 19** under Mitsunobu reaction conditions [40].

For the synthesis of monobenzyl derivatives of sulfamide, we chose one-pot two-step procedure [39], where the first step is the condensation reaction of sulfamide (compound** 6**) and aryl aldehydes and the second step is the treatment of reaction mixture with NaBH_4_, where the reduction of C=N double bond takes place. Under these conditions utilizing aldehydes** 20a**-**b**,** 17,** and** 21,** monosubstituted sulfamides** 11**,** 12**,** 14**, and** 16** were obtained (Scheme 1).

### 2.2. CA Inhibition and Structure-Activity Relationship (SAR)

Sulfamides** 11**–**16** were investigated as inhibitors of four physiologically relevant CA isoforms, the cytosolic hCAs I and II, and the transmembrane, tumor-associated hCAs IX and XII (Table 1). The following SAR can be observed from the data of Table 1.

(i) hCA I was poorly inhibited by sulfamides** 11**–**16**, which showed a compact behavior of medium-potency, weak inhibitors, with K_*I*_s ranging from 1440 to 4050 nM. Interestingly, the compounds with the bulkier tails,** 15** and** 16,** were more effective as hCA I inhibitors compared to the derivatives with the OH, OMe, or alkynyl-ether moieties** 11**–**14**. It may be observed that the standard drug acetazolamide (AAZ, a clinically used drug) was a more effective hCA I inhibitor compared to the sulfamides reported here.

(ii) The new sulfamides inhibited the physiologically dominant cytosolic isoform hCA II with K_*I*_s ranging from 9.5 to 188 nM. It is interesting to note that derivatives** 13** and** 14** were effective hCA II inhibitors (comparable to AAZ), with inhibition constants of 9.5–9.8 nM (Table 1). The two compounds incorporate the same but-2-yn-1-yloxy-tail and only differ by the presence of Schiff's base (imine) moiety in compound** 13**, which is reduced to the secondary amine in compound** 14**. It is obvious that this structural modification has a minimal effect on the hCA II inhibitory properties, whereas the nature of the tail present in position 4 of the benzene ring (with respect to the zinc-binding group) has a crucial role in their binding affinity to the enzyme. Indeed, the compounds with such smaller moieties (than the but-2-yn-1-yloxy-one), like** 11** and** 12**, but also those with larger and bulkier such tails (compounds** 15** and** 16**), were less effective CAIs compared to compounds** 13** and** 14** against hCA II. Indeed, compounds** 11** and** 16** were medium-potency hCA II inhibitors (K_*I*_s ranging from 43.3 to 74.1 nM) whereas compounds** 12** and** 15** were even weaker, with inhibition constants in the range of 134–188 nM (Table 1). The net difference of activity between compounds** 11** and** 12** which only differ by a CH_3_ moiety should be noted. In the case of the imine-amine pair** 15**,** 16**, the imine** 15** was 4.3 times a weaker hCA II inhibitor compared to the amine** 16**.

(iii) Both transmembrane isoforms, hCA IX and XII, were effectively inhibited by sulfamides** 11**–**16**, with little SAR evident from data of Table 1. Thus, for hCA IX the inhibition constants only ranged between 40.7 and 62.1 nM, whereas against hCA XII they were in the range of 5.8–8.4 nM. Thus all these sulfamides were medium-potency hCA IX inhibitors and were highly effective as hCA XII inhibitors (Table 1).

### 2.3. Crystallography

To better understand at structural level the molecular features determining the inhibition profiles of this new series of compounds against hCAs, we have solved the crystal structure of the cytosolic dominant isoform hCA II in complex with its highest affinity inhibitor (compound** 14**) in the series.

Crystals of hCA II/**14** adduct were isomorphous with those of the native protein [42], allowing for the analysis of the structure by difference Fourier techniques. Data collection and refinement statistics are shown in Table 2. Inhibitor binding did not generate major changes in the structure of hCA II as proved by the low value of the RMSD calculated by superposing the Cα atoms in the adduct and the noninhibited enzyme (0.3 Å). The overall quality of the model was high, with 88.6% of the non-glycine residues located in the allowed regions of the Ramachandran plot (Table 2).

**Figure 2:**
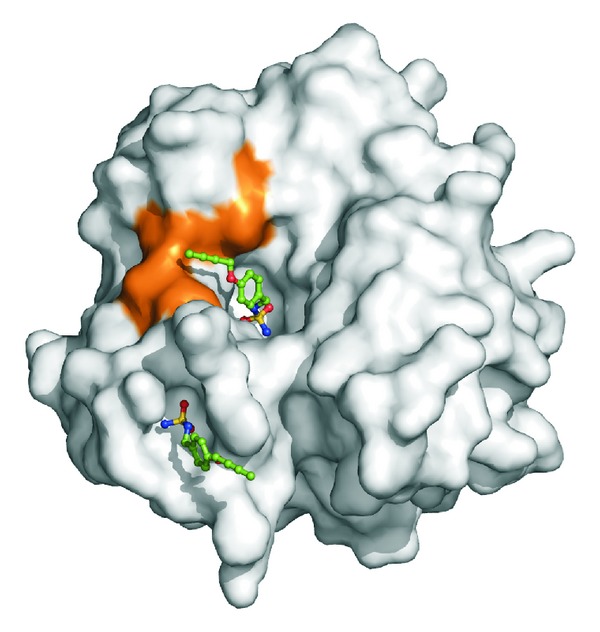
Solvent accessible surface of hCA II in its complex with** 14**. The two molecules of the inhibitor bound within the active site cavity and on the protein surface are shown in stick representation. The hydrophobic cleft defined by residues Phe131, Val135, Pro202, and Leu204 is highlighted in orange.

The inspection of the electron density maps at various stages of the crystallographic refinement revealed the binding of two inhibitor molecules: the first one on the protein surface and the second in the active site cavity (Figure 2). The binding of the inhibitor on the protein surface will not be discussed here, since it occurs far from the active site; thus it is not correlated with the inhibition properties of the molecule. On the contrary the binding of the molecule in the active site will be analyzed in detail since it is clearly associated with the high inhibitory potency of the investigated sulfamide.

**Figure 3:**
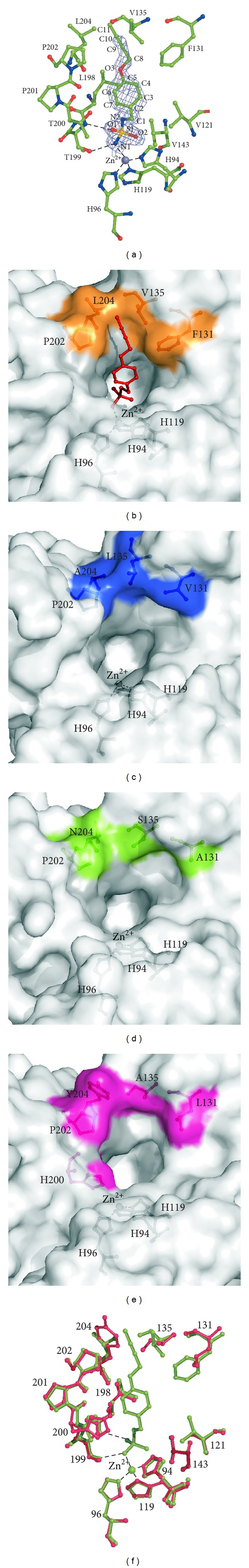
(a) Active site region of the hCA II/**14** complex. The inhibitor is shown in association with a σA-weighted |2Fo − Fc| map (at 1.0 σ). Hydrogen bonds, van der Waals interactions (distance of <4.0 Å), and the active site Zn^2+^-ion coordination are also shown. (b, c, d, and e) Solvent accessible surface of hCAs II, IX, XII, and I: the hydrophobic cleft defined by residues 131, 135, 202, and 204 is highlighted in orange (hCA II), blue (hCA IX), green (hCA XII), and magenta (hCA I). For hCA I His200 is also showed in magenta. (f) Structural superposition of the hCA I (magenta) and hCA II (green) active sites. The inhibitor** 14** is shown as bound in its complex with hCA II.

As clearly evidenced in Figure 3(a) the electron density for the molecule bound in the active-site is very well defined for the phenylmethyl sulfamide moiety and slightly less defined for the but-2-yn-1-yloxy tail indicating some flexibility of this region. The compound is anchored to the active site coordinating the catalytic Zn^2+^ ion by means of one nitrogen atom of the sulfamide group (N1) and displacing the zinc bound water molecule/hydroxide ion (Figure 3(a)), similarly to what is observed for other sulfamides (compounds** 6**–**10**) and sulfonamides/sulfamates whose crystal structures in adduct with CAs have been reported [16–18, 34, 37]. The same nitrogen atom N1 also interacts with Thr199 forming a hydrogen bond with its side chain, whereas one of sulfamide oxygen atoms forms a second hydrogen bond with the backbone nitrogen atom of the same residue (Figure 3(a)). It is interesting to note that the single bond N2-C1 adopts a trans-conformation (dihedral angle S1-N2-C1-C2 of about 175°), close to the trans-conformation expected for compound** 13**, which contains in the same position a double bond. Thus it is tempting to speculate that this behavior should be at the basis of the almost identical affinity that the two molecules show for hCA II (see Table 1).

The phenyl ring of the inhibitor resides in the middle of the active site channel, making various van der Waals interactions with the side chains of Phe131, Leu198, Pro201, and Thr200 while the but-2-yn-1-yloxy tail lies in a small hydrophobic cleft on the protein surface, defined by residues Phe131, Val135, Pro202, and Leu204 (Figure 3(b)). This cleft has already been identified as an important region in the recognition of CAIs [1, 43]. In agreement with these data, this interaction seems to have important consequences on the inhibitory properties of this series of compounds against hCA II (see Table 1); indeed, inhibitors containing the but-2-yn-1-yloxy tail (compounds** 13** and** 14**) are those with the best inhibitory properties against the enzyme, while compounds with shorter (compounds** 11** and** 12**) or bulkier (compounds** 15** and** 16**) tails have less inhibitory potency. Indeed, compounds with shorter tails probably establish less extensive interactions with this cleft, while those with bulkier tails are unable to interact with it.

A so clear correlation between the tail and the inhibition constants is not observed for the other studied isoforms. Indeed in the case of hCA IX [44] and hCA XII [45] all studied compounds, although showing good affinity for the enzymes, present a much flat inhibition profile, not correlated to the size of the tail (see Table 1). Interestingly, in both cases the aforementioned hydrophobic cleft is larger (Figures 3(c) and 3(d)) and probably does not interact opportunely with the tail and does not allow a good discrimination.

A different situation is observed in the case of hCA I for which much higher inhibition constants are observed. The structural superposition of hCA II/**14** complex with hCA I [46] (Figure 3(f)) can give a reasonable explanation of these data. Indeed, most of the residues involved in the interaction of the inhibitor with hCA II are conserved also in the isoform I. However, the substitution of Thr200 with His200 in hCA I plays an important role in destabilizing the enzyme-inhibitor interaction since this residue is much more bulky and makes the active site narrower (Figures 3(e) and 3(f)). Therefore only an important structural rearrangement of the enzyme active site could allow the binding of the inhibitor, determining the very low affinity toward hCA I, as previously observed for other hCA I/inhibitor complexes [47].

As mentioned above very few papers describing sulfamide-containing derivatives crystallized with hCA II have been reported [16–18, 37, 38]. In these adducts a very weak additional H-bond interaction is observed between the Thr200OG atom and the second nitrogen atom of the sulfamide moiety. This weak interaction is absent in our case. The finding that compound** 14** still remains a very good CA inhibitor despite this absence further confirms that such interaction does not have a great effect on the stabilization of the binding.

In conclusion, in this paper we report the X-ray structure of a new sulfamide inhibitor of CAs in complex with hCA II, together with an inhibition study of a family of structurally related compounds for the CA isoforms I, II, IX, and XII. The data reported here provide further insights into sulfamide binding mechanism confirming that this zinc-binding group could be usefully exploited for obtaining new potent and selective CAIs. In particular, the but-2-yn-1-yloxy tail emerges as a very interesting group for this purpose due to its capability to establish strong van der Waals interactions with a hydrophobic cleft on the hCA II surface delimited by residues Phe131, Val135, Pro202, and Leu204. Indeed, the complementarity of the tail with the cleft suggests that different substituents could be used to discriminate between isoforms having cleft with different sizes.

## 3. Materials and Methods

### 3.1. Chemistry

Reagents and starting materials were obtained from commercial sources and used as received. Compound** 19** was synthesized according to literature procedure [48]. The solvents were purified and dried by standard procedures prior to use; petroleum ether (PE) of boiling range 40–60°C was used. Flash chromatography was carried out using Merck silica gel (230–400 mesh). Thin-layer chromatography was performed on silica gel; spots were visualized with UV light (254 and 365 nm). Melting points were determined on an OptiMelt automated melting point system. NMR spectra were recorded on Varian Mercury (400 MHz) spectrometer with chemical shifts values (*δ*) in ppm relative to TMS using the residual DMSO-*d*_6_ signal as an internal standard. Elemental analyses were performed on a Carlo Erba CHNS-O EA-1108 apparatus.

#### 3.1.1. General Procedure for the Synthesis of 4-Alkoxy Substituted Benzaldehydes

To a mixture of 4-hydroxybenzaldehyde (**17**) (15.58 mmol), PPh_3_ (16.22 mmol), and corresponding alcohol under argon atmosphere dry DCM (100 mL) and dry THF (100 mL) were added. To this mixture at 0°C diisopropyl azodicarboxylate (DIAD) (15.76 mmol) was slowly added and reaction mixture was stirred at room temperature for 21 h. H_2_O (75 mL) and brine (15 mL) were added and the mixture was extracted with DCM (3 × 100 mL). Organic layers were combined, dried over Na_2_SO_4_, and solvent was evaporated. The crude product was purified by column chromatography on silica gel.

#### 3.1.2. 4-(But-2-yn-1-yloxy)benzaldehyde (**20a**)

Compound** 20a** was obtained from 4-hydroxybenzaldehyde (**17**) (1.90 g, 15.58 mmol), PPh_3_ (4.27 g, 16.27 mmol), but-2-yn-1-ol (**18**) (0.87 mL, 11.54 mmol), and DIAD (3.13 mL, 15.82 mmol). The crude product was purified by column chromatography (toluene) and crystallized from EtOH to yield** 20a** (1.55 g, 77%) as white solid. Mp 66–68°C.

^1^H NMR (400 MHz, DMSO-*d*_6_) *δ*: 1.84 (t, 3H, *J* = 2.3 Hz), 4.88 (q, 2H, *J* = 2.3 Hz), 7.13–7.17 (m, 2H), 7.86–7.90 (m, 2H), 9.88 (s, 1H).

^13^C NMR (100 MHz, DMSO-*d*_6_) *δ*: 3.1, 56.3, 74.1, 84.2, 115.2, 130.0, 131.7, 162.3, 191.3.

Anal. Calcd. for C_11_H_10_O_2_ (174.20): C, 75.84; H, 5.79. Found: C, 75.60; H, 5.81.

#### 3.1.3. 4-[(2-Methylquinolin-4-yl)methoxy]benzaldehyde (**20b**)

Compound** 20b** was obtained from 4-hydroxybenzaldehyde (**17**) (1.90 g, 15.58 mmol), PPh_3_ (4.27 g, 16.27 mmol), (2-methylquinolin-4-yl)methanol(**19**) [48] (2.00 g, 11.54 mmol), and DIAD (3.13 mL, 15.82 mmol). The crude product was purified by column chromatography (PE/EtOAC 3:1 then 1:1) to yield** 20b** (2.70 g, 85%) as yellow solid. Mp 98–100°C.

^1^H NMR (400 MHz, DMSO-*d*_6_) *δ*: 2.66 (s, 3H), 5.72 (s, 2H), 7.31–7.36 (m, 2H), 7.55 (s, 1H), 7.58 (t, 1H, *J* = 7.7 Hz), 7.75 (t, 1H, *J* = 7.7 Hz), 7.88–7.93 (m, 2H), 7.98 (d, 1H, *J* = 8.4 Hz), 8.10 (d, 1H, *J* = 8.4 Hz), 9.90 (s, 1H).

^13^C NMR (100 MHz, DMSO-*d*_6_) *δ*: 25.0, 66.6, 115.4, 120.2, 123.7, 123.8, 125.9, 128.9, 129.4, 130.1, 131.9, 141.5, 147.4, 158.6, 163.0, 191.3.

Anal. Calcd. for C_18_H_15_NO_2_ (277.32): C, 77.96; H, 5.45; N, 5.05. Found: C, 77.66; H, 5.47; N, 5.03.

#### 3.1.4. General Procedure for the Synthesis Benzylidene Sulfamides

To sulfamide (**6**) (3.12 mmol) glacial acetic acid (5 mL) followed by the corresponding benzaldehyde (3.12 mmol) was added. Reaction mixture was stirred at 60°C for 23 h. EtOH was added and solvent was evaporated in vacuum. The crude product was purified by column chromatography on silica gel.

#### 3.1.5. N-([4-(But-2-yn-1-yloxy)phenyl]methylidene)sulfuric Diamide (**13**)

Compound** 13** was obtained from sulfamide (**6**) (0.30 g, 3.12 mmol) and 4-(but-2-yn-1-yloxy)benzaldehyde (**20a**) (0.54 g, 3.12 mmol). The crude product was purified by column chromatography (PE/EtOAc 2 : 1) and crystallized from MeCN/H_2_O to yield** 13 (**0.32 g, 41%) as white solid. Mp 192–194°C.

^1^H NMR (400 MHz, DMSO-*d*_6_) *δ*: 1.84 (t, 3H, *J* = 2.4 Hz), 4.88 (q, 2H, *J* = 2.4 Hz), 7.12–7.17 (m, 2H), 7.29 (s, 2H), 7.93–7.98 (m, 2H), 8.83 (s, 1H).

^13^C NMR (100 MHz, DMSO-*d*_6_) *δ*: 3.2, 56.4, 74.1, 84.3, 115.6, 125.6, 132.5, 162.1, 165.7.

Anal. Calcd. for C_11_H_12_N_2_O_3_S (252.29): C, 52.37; H, 4.79; N, 11.10. Found: C, 52.03; H, 4.72; N, 11.07.

#### 3.1.6. N-({4-[(2-Methylquinolin-4-yl)methoxy]phenyl}methylidene)sulfuric Diamide (**15**)

Compound** 15** was obtained from sulfamide (**6**) (0.30 g, 3.12 mmol) and 4-[(2-methylquinolin-4-yl) methoxy]benzaldehyde (**20b**) (0.87 g, 3.12 mmol). The crude product was purified by column chromatography (PE/EtOAc 2 : 1 then neat EtOAc) and crystallized from EtOH/H_2_O to yield** 15 (**0.41 g, 37%) as white solid. Mp 99–101°C.

^1^H NMR (400 MHz, DMSO-*d*_6_) *δ*: 2.67 (s, 3H), 5.75 (s, 2H), 7.31 (s, 2H), 7.33–7.38 (m, 2H), 7.57 (s, 1H), 7.57–7.63 (m, 1H), 7.73–7.78 (m, 1H), 7.96–8.03 (m, 3H), 8.12 (d, 1H, *J* = 8.4 Hz), 8.86 (s, 1H).

^13^C NMR (100 MHz, DMSO-*d*_6_) *δ*: 25.0, 66.6, 115.8, 120.2, 123.7, 123.8, 125.7, 126.0, 128.9, 129.5, 132.7, 141.6, 147.4, 158.6, 162.8, 165.8.

Anal. Calcd. for C_18_H_17_N_3_O_3_S (355.41): C, 60.83; H, 4.82; N, 11.82. Found: C, 60.39; H, 4.87; N, 11.71.

#### 3.1.7. General One-Pot Procedure for the Synthesis of Monosubstituted Sulfamide

To sulfamide (**6**) (1 eq) glacial acetic acid followed by the corresponding benzaldehyde (1 eq) was added. Reaction mixture was stirred at 60°C for 23 h. NaBH_4_ (10 eq) portionwise was added followed by extra glacial acetic acid. Reaction mixture was stirred at room temperature for 18 h before it was quenched with sat. aq. NH_4_Cl. EtOH was added and solvent was evaporated in vacuum. H_2_O was added and mixture was extracted with EtOAc. Combined organic layers were dried over Na_2_SO_4_ and purified by column chromatography on silica gel.

#### 3.1.8. N-(4-Hydroxybenzyl)sulfuric Diamide (**11**)

Compound** 11** was obtained from sulfamide (**6**) (0.30 g, 3.12 mmol), 4-hydroxybenzaldehyde (**17**) (0.38 g, 3.12 mmol) in AcOH (5 mL), and NaBH_4_ (1.18 g, 31.2 mmol) with extra AcOH (15 mL). Reaction mixture was quenched with sat. aq. NH_4_Cl (15 mL), diluted with H_2_O (40 mL), and extracted with EtOAc (3 × 30 mL). The crude product was purified by column chromatography (PE/EtOAc 1 : 1) to yield** 11** (0.10 g, 15%) as white solid. Mp 140–142°C.

^1^H NMR (400 MHz, DMSO-*d*_6_) *δ*: 3.94 (d, 2H, *J* = 6.5 Hz), 6.55 (s, 2H), 6.67–6.72 (m, 2H), 6.84 (t, 1H, *J* = 6.5 Hz), 7.10–7.15 (m, 2H), 9.28 (s, 1H).

^13^C NMR (100 MHz, DMSO-*d*_6_) *δ*: 45.8, 114.9, 128.7, 129.0, 156.4.

Anal. Calcd. for C_7_H_10_N_2_O_3_S (202.23): C, 41.57; H, 4.98; N, 13.85. Found: C, 41.02; H, 5.07; N, 13.67.

#### 3.1.9. N-(4-Methoxybenzyl)sulfuric Diamide (**12**)

Compound** 12** was obtained from sulfamide (**6**) (0.30 g, 3.12 mmol), 4-methoxybenzaldehyde (**21**) (0.38 mL, 3.12 mmol) in AcOH (5 mL), and NaBH_4_ (1.18 g, 31.2 mmol) with extra AcOH (15 mL). Reaction mixture was quenched with sat. aq. NH_4_Cl (3 mL), diluted with H_2_O (50 mL), and extracted with EtOAc (3 × 30 mL). The crude product was purified by column chromatography (PE/EtOAc 2 : 1) and crystallized from EtOH/H_2_O to yield** 12 **(0.27 g, 40%) as white solid. Mp 118–120°C.

^1^H NMR (400 MHz, DMSO-*d*_6_) *δ*: 3.73 (s, 3H), 4.00 (d, 2H, *J* = 6.5 Hz), 6.88 (s, 2H), 6.85–6.90 (m, 2H), 6.93 (t, 1H, *J* = 6.5 Hz), 7.23–7.28 (m, 2H).

^13^C NMR (100 MHz, DMSO-*d*_6_) *δ*: 45.6, 55.1, 113.6, 129.0, 130.5, 158.3.

Anal. Calcd. for C_8_H_12_N_2_O_3_S (216.26): C, 44.43; H, 5.59; N, 12.95. Found: C, 44.54; H, 5.52; N, 12.81.

#### 3.1.10. N-[4-(But-2-yn-1-yloxy)benzyl]sulfuric Diamide (**14**)

Compound** 14** was obtained from sulfamide (**6**) (0.26 g, 2.67 mmol), 4-(but-2-yn-1-yloxy)benzaldehyde (**20a**) (0.47 g, 2.67 mmol) in AcOH (5 mL), and NaBH_4_ (1.01 g, 26.7 mmol) with extra AcOH (15 mL). Reaction mixture was quenched with sat. aq. NH_4_Cl (15 mL), diluted with H_2_O (40 mL), and extracted with EtOAc (3 × 30 mL). The crude product was purified by column chromatography (PE/EtOAc 2 : 1 then 1 : 1) and crystallized from DCM to yield** 14 **(0.04 g, 9%) as white solid. Mp 93–95°C.

^1^H NMR (400 MHz, DMSO-*d*_6_) *δ*: 1.82 (t, 3H, *J* = 2.3 Hz), 4.00 (d, 2H, *J* = 6.3 Hz), 4.71 (q, 2H, *J* = 2.3 Hz), 6.59 (s, 2H), 6.88–6.97 (m, 3H), 7.23–7.28 (m, 2H).

^13^C NMR (100 MHz, DMSO-*d*_6_) *δ*: 3.1, 45.6, 55.8, 74.8, 83.4, 114.4, 128.9, 131.1, 156.4.

Anal. Calcd. for C_11_H_14_N_2_O_3_S (254.31): C, 51.95; H, 5.55; N, 11.02. Found: C, 51.82; H, 5.56; N, 10.97.

#### 3.1.11. N-{4-[(2-Methylquinolin-4-yl)methoxy]benzyl}sulfuric Diamide (**16**)

Compound** 16** was obtained from sulfamide (**6**) (0.17 g, 1.80 mmol), 4-[(2-methylquinolin-4-yl) methoxy]benzaldehyde (**20b**) (0.50 g, 1.80 mmol) in AcOH (5 mL), and NaBH_4_ (0.68 g, 18.0 mmol) with extra AcOH (15 mL). Reaction mixture was quenched with sat. aq. NH_4_Cl (10 mL), diluted with H_2_O (40 mL), and extracted with EtOAc (3 × 30 mL). The crude product was purified by column chromatography (PE/EtOAc 1 : 2) to yield** 16 **(0.14 g, 21%) as yellow solid. Mp 180-181°C.

^1^H NMR (400 MHz, DMSO-*d*_6_) *δ*: 2.66 (s, 3H), 4.02 (d, 2H, *J* = 6.4 Hz), 5.60 (s, 2H), 6.60 (s, 2H), 6.97 (t, 1H, *J* = 6.4 Hz), 7.07–7.12 (m, 2H), 7.28–7.33 (m, 2H), 7.55 (s, 1H), 7.56–7.61 (m, 1H), 7.72–7.77 (m, 1H), 7.97 (d, 1H, *J* = 8.4 Hz), 8.10 (d, 1H, *J* = 8.4 Hz).

^13^C NMR (100 MHz, DMSO-*d*_6_) *δ*: 25.0, 45.6, 66.2, 114.6, 120.1, 123.7, 123.9, 125.9, 128.9, 129.1, 129.4, 131.3, 142.4, 147.4, 157.1, 158.6.

Anal. Calcd. for C_18_H_19_N_3_O_3_S (357.43): C, 60.49; H, 5.36; N, 11.76. Found: C, 60.37; H, 5.38; N, 11.81.

### 3.2. CA Inhibition Assays

A stopped-flow CO_2_ hydration assay with an Applied Photophysics instrument was used for measuring the inhibition of hCAs I, II, IX, and XII by the new compounds reported here. Phenol red (at a concentration of 0.2 mM) has been used as indicator, working at the absorbance maximum of 557 nm, with 20 mM Hepes (pH 7.4) or 20 mM Tris (pH 8.3) as buffers, and 20 mM Na_2_SO_4_ or NaClO_4_ (for maintaining the ionic strength constant). The initial rates of the CA-catalyzed CO_2_ hydration reaction were followed for a period of 10–100 s [41]. The concentrations of substrate (CO_2_) ranged from 1.7 to 17 mM for the determination of the inhibition constants, with at least six traces of the initial 5–10% of the reaction being used for determining the initial velocity, for each inhibitor. The uncatalyzed rates were determined and subtracted from the total observed rates. Stock solutions of inhibitors (10 mM) were prepared in distilled-deionized water and dilutions up to 0.01 nM were done with the assay buffer. Enzyme and inhibitor solutions were preincubated prior to assay for 15 min (at room temperature), in order to allow for the formation of the E-I complex. The inhibition constants were obtained by nonlinear least-squares methods using PRISM 3 and the Cheng-Prusoff equation as reported earlier by our groups. The kinetic parameters for the uninhibited enzymes were derived from Lineweaver-Burk plots, as reported earlier [49–51], and represent the mean from at least three different determinations.

### 3.3. X-Ray Studies

hCA II/**14** complex was obtained by adding a 5-molar excess of inhibitor to a 10 mg/mL protein solution in 20 mM Tris-HCl pH 8, 0.1% DMSO. Crystals of the complex were obtained using the hanging drop vapor diffusion technique. In particular 2 *μ*L of complex solution and 2 *μ*L of precipitant solution (1.4 M Na-Citrate, 100 mM Tris-HCl pH 8.0) were mixed and suspended over a reservoir containing 1 mL of precipitant solution at 20°C. X-ray diffraction data were collected at 100 K, using a copper rotating anode generator developed by Rigaku and equipped with a Rigaku Saturn CCD detector. Prior to cryogenic freezing, the crystals were transferred to the precipitant solution with the addition of 15% (v/v) glycerol. Data were processed using the HKL2000 package [52]. Diffraction data were indexed in the P2_1_ space group with one molecule in the asymmetric unit. Unit cell parameters and data reduction statistics are reported in Table 2. The atomic coordinates of hCA II (PDB entry 1CA2) [42] were used as a starting model for crystallographic refinement after deletion of non-protein atoms. Structure refinement (in the 20.0–1.85 Å resolution range) was carried out using CNS [53] and model building was performed with O [54]. Inhibitor molecules were identified from peaks in |Fo| *‒* |Fc| maps and gradually built into the model over several rounds of refinement. Restraints on inhibitor bond angles and distances were taken from similar structures in the Cambridge Structural Database [55] whereas standard restraints were used on protein bond angles and distances throughout refinement. The correctness of stereochemistry was finally checked using PROCHECK [56]. Final refinement statistics are presented in Table 2. The atomic coordinates of hCA II/**14** complex were deposited in the Protein Data Bank, accession code 4PQ7.

## Figures and Tables

**Table 1:** hCAs I, II, IX, and XII inhibition data with sulfamides **11–16**. Acetazolamide (AAZ) has been used as standard drug. Analyses were performed with a CO_2_ hydrase, stopped-flow assay [41].

Compounds	*K*_*I*_ (nM)∗			
	hCA I	hCA II	hCA IX	hCA XII
**11**	2180	74.1	40.7	5.8
**12**	4050	134	60.0	6.6
**13**	1940	9.8	59.1	8.4
**14**	1810	9.5	61.7	8.1
**15**	1650	188	56.3	6.5
**16**	1440	43.3	62.1	6.6
AAZ	250	12	25	5.7

∗Mean from 3 different assays, errors in the range of ±10% of the reported values.

**Table 2:** Crystal parameters, data collection, and refinement statistics.

Crystal parameters	
Space group	P2_1_
*a* (Å)	42.4
*b* (Å)	41.3
*c* (Å)	71.8
*γ* (°)	104.3
Number of independent molecules	1

Data collection statistics	
Resolution (Å)	50−1.85
Wavelength (Å)	1.54178
Temperature (K)	100
*R*_merge_ (%)^a^	3.4 (7.1)
Mean *I*/*σ*(*I*)	35.8 (13.6)
Total reflections	80810
Unique reflections	20026
Redundancy (%)	4.0 (2.5)
Completeness (%)	96.1 (84.8)

Refinement statistics	
*R*_factor_ (%)^b^	15.6
*R*_free_ (%)^b^	19.6
RMSD from ideal geometry	
Bond lengths (Å)	0.012
Bond angles (°)	1.7
Number of protein atoms	2091
Number of water molecules	215
Number of inhibitor atoms (2 molecules)	34
Average B factor (Å^2^)	
All atoms	12.6
Protein atoms	11.6
Inhibitor 1 atoms	19.9
Inhibitor 2 atoms	27.1
Water molecules	20.6
Ramachandran plot	
Residues in the most favored regions (%)	88.6
Residues in additional allowed regions (%)	11.0
Residues in generously allowed regions (%)	0.5

^a^ *R*_merge_ = ∑_*hkl*_∑_*i*_|*I*_*i*_(*hkl*) − 〈*I*(*hkl*)〉 | /∑_*hkl*_∑_*i*_ *I*_*i*_(*hkl*), where *I*_*i*_(*hkl*) is the intensity of an observation and 〈*I*(*hkl*)〉 is the mean value for its unique reflection; summations are over all reflections.

^b^ *R*_factor_ = ∑_*h*_||Fo(*h*)|−|Fc(*h*)||/∑_*h*_|Fo(*h*)|, where Fo and Fc are the observed and calculated structure-factor amplitudes, respectively. *R*_free_ is calculated in same manner as *R*_factor_, except that it uses 5% of the data omitted from refinement.

## References

[1] Alterio V, di Fiore A, D'Ambrosio K, Supuran CT, de Simone G (2012). Multiple binding modes of inhibitors to carbonic anhydrases: how to design specific drugs targeting 15 different isoforms?. *Chemical Reviews*.

[2] Supuran CT (2008). Carbonic anhydrases: novel therapeutic applications for inhibitors and activators. *Nature Reviews Drug Discovery*.

[3] Supuran CT, Scozzafava A (2007). Carbonic anhydrases as targets for medicinal chemistry. *Bioorganic and Medicinal Chemistry*.

[4] Supuran CT, Winum J-Y (2009). Carbonic anhydrases as drug targets—general presentation. *Drug Design of Zinc-Enzyme Inhibitors: Functional, Structural, and Disease Applications*.

[5] Winum J-Y, Rami M, Scozzafava A, Montero J-L, Supuran CT (2008). Carbonic anhydrase IX: a new druggable target for the design of antitumor agents. *Medicinal Research Reviews*.

[6] Supuran CT, Scozzafava A, Casini A (2003). Carbonic anhydrase inhibitors. *Medicinal Research Reviews*.

[7] Winum J, Vullo D, Casini A, Montero J, Scozzafava A, Supuran CT (2003). Carbonic anhydrase inhibitors. Inhibition of cytosolic isozymes I and II and transmembrane, tumor-associated isozyme IX with sulfamates including EMATE also acting as steroid sulfatase inhibitors. *Journal of Medicinal Chemistry*.

[8] Winum J, Vullo D, Casini A, Montero J, Scozzafava A, Supuran CT (2003). Carbonic anhydrase inhibitors: inhibition of transmembrane, tumor-associated isozyme IX, and cytosolic isozymes I and II with aliphatic sulfamates. *Journal of Medicinal Chemistry*.

[9] Winum J-Y, Scozzafava A, Montero J-L, Supuran CT (2006). Therapeutic potential of sulfamides as enzymes inhibitors. *Medicinal Research Reviews*.

[10] Winum J-Y, Scozzafava A, Montero J-L, Supuran CT (2006). The sulfamide motif in the design of enzyme inhibitors. *Expert Opinion on Therapeutic Patents*.

[11] Scozzafava A, Banciu MD, Popescu A, Supuran CT (2000). Carbonic anhydrase inhibitors: inhibition of isozymes I, II and IV by sulfamide and sulfamic acid derivatives. *Journal of Enzyme Inhibition and Medicinal Chemistry*.

[12] Casini A, Winum J, Montero J, Scozzafava A, Supuran CT (2003). Carbonic anhydrase inhibitors: inhibition of cytosolic isozymes I and II with sulfamide derivatives. *Bioorganic and Medicinal Chemistry Letters*.

[13] Casini A, Antel J, Abbate F (2003). Carbonic anhydrase inhibitors: SAR and X-ray crystallographic study for the interaction of sugar sulfamates/sulfamides with isozymes I, II and IV. *Bioorganic and Medicinal Chemistry Letters*.

[14] Winum J, Innocenti A, Nasr J (2005). Carbonic anhydrase inhibitors: Synthesis and inhibition of cytosolic/tumor-associated carbonic anhydrase isozymes I, II, IX, and XII with *N*-hydroxysulfamides—a new zinc-binding function in the design of inhibitors. *Bioorganic and Medicinal Chemistry Letters*.

[15] Winum J-Y, Cecchi A, Montero J-L, Innocenti A, Scozzafava A, Supuran CT (2005). Carbonic anhydrase inhibitors. Synthesis and inhibition of cytosolic/tumor-associated carbonic anhydrase isozymes I, II, and IX with boron-containing sulfonamides, sulfamides, and sulfamates: toward agents for boron neutron capture therapy of hypoxic tumors. *Bioorganic and Medicinal Chemistry Letters*.

[16] Abbate F, Supuran CT, Scozzafava A, Orioli P, Stubbs MT, Klebe G (2002). Nonaromatic sulfonamide group as an ideal anchor for potent human carbonic anhydrase inhibitors: role of hydrogen-bonding networks in ligand binding and drug design. *Journal of Medicinal Chemistry*.

[17] Winum J, Temperini C, El Cheikh K (2006). Carbonic anhydrase inhibitors: clash with Ala65 as a means for designing inhibitors with low affinity for the ubiquitous isozyme II, exemplified by the crystal structure of the topiramate sulfamide analogue. *Journal of Medicinal Chemistry*.

[18] Temperini C, Winum J, Montero J, Scozzafava A, Supuran CT (2007). Carbonic anhydrase inhibitors: The X-ray crystal structure of the adduct of N-hydroxysulfamide with isozyme II explains why this new zinc binding function is effective in the design of potent inhibitors. *Bioorganic and Medicinal Chemistry Letters*.

[19] Marquis RE, Whitson JT (2005). Management of glaucoma: Focus on pharmacological therapy. *Drugs and Aging*.

[20] Vullo D, Innocenti A, Nishimori I (2005). Carbonic anhydrase inhibitors. Inhibition of the transmembrane isozyme XII with sulfonamides—a new target for the design of antitumor and antiglaucoma drugs?. *Bioorganic and Medicinal Chemistry Letters*.

[21] Supuran CT, Casini A, Scozzafava A, Supuran CT, Scozzafava A, Conway J (2004). Development of sulfonamide carbonic anhydrase inhibitors (CAIs). *Carbonic Anhydrase—Its Inhibitors and Activators*.

[22] Fabrizi F, Mincione F, Somma T (2012). A new approach to antiglaucoma drugs: carbonic anhydrase inhibitors with or without NO donating moieties. Mechanism of action and preliminary pharmacology. *Journal of Enzyme Inhibition and Medicinal Chemistry*.

[23] Loiselle FB, Morgan PE, Alvarez BV, Casey JR (2004). Regulation of the human NBC_3_ Na^+^/HCO_3_
^−^ cotransporter by carbonic anhydrase II and PKA. *The American Journal of Physiology—Cell Physiology*.

[24] Rivera C, Voipio J, Kaila K (2005). Two developmental switches in GABAergic signalling: the K^+^-Cl^−^ cotransporter KCC2 and carbonic anhydrase CAVII. *Journal of Physiology*.

[25] Vullo D, Voipio J, Innocenti A (2005). Carbonic anhydrase inhibitors. Inhibition of the human cytosolic isozyme VII with aromatic and heterocyclic sulfonamides. *Bioorganic and Medicinal Chemistry Letters*.

[26] Lyons KE, Pahwa R, Comella CL (2003). Benefits and risks of pharmacological treatments for essential tremor. *Drug Safety*.

[27] Hori K, Ishida S, Inoue M (2004). Treatment of cystoid macular edema with oral acetazolamide in a patient with best vitelliform macular dystrophy. *Retina*.

[28] Supuran CT (2003). Carbonic anhydrase inhibitors in the treatment and prophylaxis of obesity. *Expert Opinion on Therapeutic Patents*.

[29] Winum JY, Scozzafava A, Montero JL, Supuran CT (2005). Sulfamates and their therapeutic potential. *Medicinal Research Reviews*.

[30] Švastová E, Hulíková A, Rafajová M (2004). Hypoxia activates the capacity of tumor-associated carbonic anhydrase IX to acidify extracellular pH. *FEBS Letters*.

[31] Splendiani G, Condò S (2006). Diuretic therapy in heart failure. *Giornale Italiano di Nefrologia*.

[32] Gadde KM, Allison DB, Ryan DH (2011). Effects of low-dose, controlled-release, phentermine plus topiramate combination on weight and associated comorbidities in overweight and obese adults (CONQUER): a randomised, placebo-controlled, phase 3 trial. *The Lancet*.

[33] Brechue WF, Maren TH (1993). A comparison between the effect of topical and systemic carbonic anhydrase inhibitors on aqueous humor secretion. *Experimental Eye Research*.

[34] Alterio V, di Fiore A, D’Ambrosio K, Supuran CT, de Simone G, Supuran CT, Winum JY (2009). X-ray crystallography of CA inhibitors and its importance in drug design. *Drug Design of Zinc-Enzyme Inhibitors: Functional, Structural, and Disease Applications*.

[35] Maryanoff BE, McComsey DF, Costanzo MJ, Hochman C, Smith-Swintosky V, Shank RP (2005). Comparison of sulfamate and sulfamide groups for the inhibition of carbonic anhydrase-II by using topiramate as a structural platform. *Journal of Medicinal Chemistry*.

[36] Klinger AL, McComsey DF, Smith-Swintosky V, Shank RP, Maryanoff BE (2006). Inhibition of carbonic anhydrase-II by sulfamate and sulfamide groups: An investigation involving direct thermodynamic binding measurements. *Journal of Medicinal Chemistry*.

[37] Di Fiore A, Monti SM, Innocenti A, Winum J, de Simone G, Supuran CT (2010). Carbonic anhydrase inhibitors: crystallographic and solution binding studies for the interaction of a boron-containing aromatic sulfamide with mammalian isoforms I-XV. *Bioorganic and Medicinal Chemistry Letters*.

[38] Rami M, Dubois L, Parvathaneni N-K (2013). Hypoxia-targeting carbonic anhydrase IX inhibitors by a new series of nitroimidazole-sulfonamides/sulfamides/sulfamates. *Journal of Medicinal Chemistry*.

[39] Abdel-Magid AF, Mehrman SJ (2006). Process for preparation of sulfamide derivatives. *Patent Cooperation Treaty International Application*.

[40] Mitsunobu O (1981). The use of diethyl azodicarboxylate and triphenylphosphine in synthesis and transformation of natural products. *Synthesis*.

[41] Khalifah RG (1971). The carbon dioxide hydration activity of carbonic anhydrase. I. Stop-flow kinetic studies on the native human isoenzymes B and C.. *The Journal of Biological Chemistry*.

[42] Eriksson AE, Jones TA, Liljas A (1988). Refined structure of human carbonic anhydrase II at 2.0 Å resolution. *Proteins: Structure, Function and Genetics*.

[43] Pacchiano F, Aggarwal M, Avvaru BS (2010). Selective hydrophobic pocket binding observed within the carbonic anhydrase II active site accommodate different 4-substituted-ureido-benzenesulfonamides and correlate to inhibitor potency. *Chemical Communications*.

[44] Alterio V, Hilvo M, Di Fiore A (2009). Crystal structure of the catalytic domain of the tumor-associated human carbonic anhydrase IX. *Proceedings of the National Academy of Sciences of the United States of America*.

[45] Whittington DA, Waheed A, Ulmasov B (2001). Crystal structure of the dimeric extracellular domain of human carbonic anhydrase XII, a bitopic membrane protein overexpressed in certain cancer tumor cells. *Proceedings of the National Academy of Sciences of the United States of America*.

[46] Kannan KK, Ramanadham M, Jones TA (1984). Structure, refinement, and function of carbonic anhydrase isozymes: Refinement of human carbonic anhydrase I. *Annals of the New York Academy of Sciences*.

[47] Alterio V, Monti SM, Truppo E, Pedone C, Supuran CT, de Simone G (2010). The first example of a significant active site conformational rearrangement in a carbonic anhydrase-inhibitor adduct: the carbonic anhydrase I-topiramate complex. *Organic and Biomolecular Chemistry*.

[48] Wang Y, Papamichelakis M, Chew W (2008). Development of a suitable process for the preparation of a TNF-α converting enzyme inhibitor, WAY-281418. *Organic Process Research and Development*.

[49] Joseph P, Turtaut F, Ouahrani-Bettache S (2010). Cloning, characterization, and inhibition studies of a *β*-carbonic anhydrase from brucella suis. *Journal of Medicinal Chemistry*.

[50] Pan P, Rodrigues GC, Scozzafava A (2013). Cloning, characterization, and sulfonamide and thiol inhibition studies of an α-carbonic anhydrase from *Trypanosoma cruzi*, the causative agent of Chagas disease. *Journal of Medicinal Chemistry*.

[51] Minakuchi M, Nishimori I, Vullo D, Scozzafava A, Supuran CT (2009). Molecular cloning, characterization, and inhibition studies of the Rv1284 beta-carbonic anhydrase from Mycobacterium tuberculosis with sulfonamides and a sulfamate. *Journal of Medicinal Chemistry*.

[52] Otwinowski Z, Minor W (1997). Processing of X-ray diffraction data collected in oscillation mode. *Methods in Enzymology*.

[53] Brünger AT, Adams PD, Clore GM (1998). Crystallography & NMR system: a new software suite for macromolecular structure determination. *Acta Crystallographica Section D*.

[54] Jones TA, Zou JY, Cowan SW (1991). Improved methods for building protein models in electron density maps and the location of errors in these models. *Acta Crystallographica A: Foundations of Crystallography*.

[55] Allen FH (2002). The Cambridge Structural Database: a quarter of a million crystal structures and rising. *Acta Crystallographica Section B: Structural Science*.

[56] Laskowski RA, MacArthur MW, Moss DS, Thornton JM (1993). PROCHECK: a program to check the stereochemical quality of protein structures. *Journal of Applied Crystallography*.

